# Patterns of Response to Methylphenidate Administration in Children with ADHD: A Personalized Medicine Approach through Clustering Analysis

**DOI:** 10.3390/children8111008

**Published:** 2021-11-04

**Authors:** Silvia Grazioli, Eleonora Rosi, Maddalena Mauri, Alessandro Crippa, Federica Tizzoni, Arianna Tarabelloni, Filippo Maria Villa, Federica Chiapasco, Maria Reimers, Erika Gatti, Silvana Bertella, Massimo Molteni, Maria Nobile

**Affiliations:** 1 Child Psychopathology Unit, Scientific Institute, IRCCS Eugenio Medea, Bosisio Parini, 23842 Lecco, Italy; silvia.grazioli@lanostrafamiglia.it (S.G.); maddalena.mauri@lanostrafamiglia.it (M.M.); alessandro.crippa@lanostrafamiglia.it (A.C.); federica.tizzoni@lanostrafamiglia.it (F.T.); arianna.tarabelloni@lanostrafamiglia.it (A.T.); filippomaria.villa@lanostrafamiglia.it (F.M.V.); erika.gatti@lanostrafamiglia.it (E.G.); silvana.bertella@lanostrafamiglia.it (S.B.); massimo.molteni@lanostrafamiglia.it (M.M.); maria.nobile@lanostrafamiglia.it (M.N.); 2 PhD in Neuroscience, School of Medicine and Surgery, University of Milano-Bicocca, 20126 Milan, Italy; 3 MD Course in Medicine and Surgery, Humanitas University, Via Manzoni 56, 20089 Milan, Italy; chiapascofederica@gmail.com (F.C.); mariasreimers@gmail.com (M.R.)

**Keywords:** attention-deficit/hyperactivity disorder, methylphenidate, near-infrared spectroscopy, machine learning, clustering, personalized medicine

## Abstract

Individual responses to methylphenidate (MPH) can significantly differ in children with attention-deficit/hyperactivity disorder (ADHD) in terms of the extent of clinical amelioration, optimal dosage needed, possible side effects, and short- and long-term duration of the benefits. In the present repeated-measures observational study, we undertook a proof-of-concept study to determine whether clustering analysis could be useful to characterize different clusters of responses to MPH in children with ADHD. We recruited 33 children with ADHD who underwent a comprehensive clinical, cognitive, and neurophysiological assessment before and after one month of MPH treatment. Symptomatology changes were assessed by parents and clinicians. The neuropsychological measures used comprised pen-and-paper and computerized tasks. Functional near-infrared spectroscopy was used to measure cortical hemodynamic activation during an attentional task. We developed an unsupervised machine learning algorithm to characterize the possible clusters of responses to MPH in our multimodal data. A symptomatology improvement was observed for both clinical and neuropsychological measures. Our model identified distinct clusters of amelioration that were related to symptom severity and visual-attentional performances. The present findings provide preliminary evidence that clustering analysis can potentially be useful in identifying different responses to MPH in children with ADHD, highlighting the importance of a personalized medicine approach within the clinical framework.

## 1. Introduction

Attention-deficit/hyperactivity disorder (ADHD) is a childhood-onset neurodevelopmental disorder that is characterized by a persistent behavioral and cognitive pattern that includes inattention, motor hyperactivity, and impulsivity symptoms that manifest inconsistently with age or developmental level [1,2]. ADHD is one of the most commonly diagnosed neurodevelopmental disorders, affecting between 2 and 7% of children and adolescents worldwide, with an average prevalence of approximately 5% [3,4]. According to a recent review, the pooled prevalence of ADHD diagnosis in Italian children aged 5–17 was 2.9% (range: 1.1–16.7%), whereas in studies based on symptoms criteria, the average estimated prevalence was 5.9% (range: 1.4 to 16.7%) [5].

Treatment strategies for ADHD include the combination of cognitive-behavioral therapy and pharmacological treatment; the latter is recommended for children aged 6–11 years and preferred for children aged 12 years and older [6]. The first-line pharmacological treatment for severe ADHD is stimulants, particularly methylphenidate (MPH) [7,8]. MPH regulates the levels of catecholamines (i.e., dopamine and norepinephrine), which are the neurotransmitters that are involved in prefrontal cortex (PFC) functions that are responsible for the maintenance of attention and cognitive control [9]. At a functional level, MPH allosterically blocks the catecholamine transporters, thus inhibiting their presynaptic reuptake [10].

Around 70% of children with ADHD show a clinically significant positive response to MPH medication [11,12]. Hundreds of randomized controlled trials demonstrate that MPH treatment is, in general, effective, safe, and well-tolerated [13,14]. The average improvements following MPH treatment, in ADHD core symptomatology and other associated manifestations (oppositional defiant, conduct, and social behavior problems), have been reported by clinicians, parents, and teachers, with a large mean effect size of about 1.41 [15,16]. However, several psychopharmacology research results highlighted that individual responses to MPH can significantly differ in terms of the extent of clinical amelioration, optimal dosage needed, possible side effects, and both short- and long-term duration of the benefits [15,17]. These pharmacological effects, especially linked to MPH treatment, were analyzed from different angles, such as the patient’s clinical characteristics, IQ, symptom presentation, disorder severity, and biomarkers, such as those from neuroimaging [18].

Accordingly, over the last two decades, the psychopharmacological research field has seen rapid growth in functional near-infrared spectroscopy (fNIRS) as a tool to monitor functional brain activity in children with neurodevelopmental conditions and to explore possible relationships between pharmacological improvement and neurophysiological activation. fNIRS has several advantages over other neuroimaging modalities, especially for children with ADHD, since it is harmless, tolerant to body movements, and portable [19]. Interestingly, fNIRS also represents a promising tool to investigate possible cortical markers of MPH treatment in ADHD. A recent review on PFC activation after MPH treatment showed that fNIRS is an effective indicator of hemodynamic response to pharmacotherapy; the reviewed studies highlighted the presence of increased oxygenated hemoglobin concentrations, with a higher frequency of right-hemisphere lateralization effects [20]. Previous studies further demonstrated that a reduced right inferior frontal gyrus and middle frontal gyrus activation in ADHD patients compared with typically developing children can be eased using a single-dose MPH administration [21,22]. However, these recent studies using fNIRS technology applied heterogeneous methods and no standardized signal processing pipelines [20,23].

The above-mentioned research evidence suggests that, currently, there is an urgent need to address individual characteristics that are associated with the response to MPH. Personalized medicine can represent a useful approach to meet the needs of each patient, to identify specific treatment plans that are based on possible clusters of response to intervention, and, in conclusion, to reduce the long-term costs of mental health [24]. Within this framework, increasing research efforts have been made recently to identify the possible behavioral and biological specificities that are linked to MPH efficacy [25,26].

Within the personalized medicine field, recent studies have applied machine learning (ML) algorithms to predict treatment outcomes (in the supervised framework) or to detect clinical sample characteristics (in the unsupervised area). ML is an ensemble of traditional statistical procedures and computer algorithms that are increasingly used as a useful tool for identifying treatment response characteristics in health care applications [27,28]. Specifically, the supervised framework in ML uses a sample of subjects that are characterized by a set of features and labels (i.e., class membership) to predict class membership in new instances based on the feature characteristics [29]. On the other hand, unsupervised ML uses a set of unlabeled features that describe the sample subjects to discover hidden data subgroups through a bottom-up approach [29]. A specific unsupervised ML problem is known as clustering [30,31], which consists of assigning labels (i.e., cluster membership) to elements of a dataset based on how similar they are to each other. Instances (subjects) who “look alike” regarding the considered features will fall into a homogeneous cluster, whereas sample subjects who are not similar will fall into one or more different clusters, based on specific distance metrics [32].

Through a bivariate model-based clustering analysis, Reimherr and colleagues [33] found no differences in response to MPH (evaluated both by self- and clinician-reported measures) in two groups of adults with ADHD, one with the ADHD-inattentive subtype and one with the ADHD-emotional dysregulation subtype. Using a support vector machine algorithm, Kim and colleagues [34] identified a multidimensional (demographical, cognitive, genetics, environmental) ensemble of variables that discriminate responders versus nonresponders on the basis of the clinician-rated Clinical Global Impressions-Improvement scale. Wong and colleagues [27] combined existing knowledge in the literature about clinical and demographic characteristics to predict the response to ADHD treatment using “learning in the model space”. The model, which is based on sociodemographic and clinical data, was capable of predicting the minimum dosage of medication required to have a chance of achieving symptomatic remission for each individual.

Despite some studies using an ML approach, the unsupervised, bottom-up framework in psychiatry is still barely explored; indeed, an ML model that addresses the specificities that are detected in response to MPH is not currently available. Further progress in this field could provide support for effective clinical decisions through a personalized treatment approach.

Given those premises, the aim of our study was to evaluate the existence of clusters of clinical, neuropsychological, and neurophysiological responses to MPH treatment in children with ADHD after a period of standardized pharmacological intervention. Particularly, we aimed to evaluate the response to MPH therapy for ADHD children through a bottom-up approach, without an a priori selection of treatment outcome measures. To achieve this aim, we implemented an unsupervised algorithm (finite Gaussian mixture model (FGMM)) for clustering [35]. We expected a heterogeneous modification in clinical and neuropsychological presentation and, possibly, in brain hemodynamic activation after MPH treatment.

## 2. Materials and Methods

The present work is a repeated-measures observational study representing the extension of a cross-sectional observational work that was previously published by our research group [36]. We recruited drug-naïve children with ADHD with a high Clinical Global Impression-Severity (CGI-S) score (≥4) [37], who received an MPH prescription after being admitted to our institute’s Child Psychopathology Unit. Subjects taking immediate-release MPH were assessed weekly. Doses were adjusted based on the treatment response and tolerability, according to Italian prescription guidelines for children and adolescents. After the second week, the doses were maintained until the second evaluation of the study (T1). Our protocol was approved by our institute’s ethics committee, “Comitato Etico IRCCS E. Medea—Sezione Scientifica Associazione La Nostra Famiglia”, in accordance with the Declaration of Helsinki (1989). Written informed consent and assent were obtained from all caregivers and participants.

### 2.1. Study Design

The study design is depicted in Table 1. The evaluations were performed during two time waves: T0 (one week before MPH intake) and T1 (after one month of continuous MPH intake). Study participation at T0 was also proposed to a control group of typically developing children (TD) for comparison purposes.

#### 2.1.1. Participants

A sample of thirty-three drug-naïve children with ADHD between the ages of 6 and 16 years (mean age = 11 ± 3.2 years) was recruited. For all patients, the ADHD diagnosis was made according to the DSM-5 criteria [1] by a child neuropsychiatrist with experience in ADHD. A child psychologist (M.Ma.) independently confirmed the diagnosis through direct clinical observation and administration of the Development and Well-Being Assessment (DAWBA) semi-structured interview with parents [38]. Thirty-one percent of patients exhibited ADHD with a predominantly inattentive presentation, 11% with a predominantly hyperactive/impulsive presentation, and 58% with a combined presentation.

The control group was composed of twenty-seven TD children that were recruited by local pediatricians and from schools in the same areas of ADHD children to be age- and gender-matched with the clinical group. The presence of psychiatric disorders was excluded using the DAWBA parent diagnostic interview.

The exclusion criteria included the presence of intellectual disability, neurological diseases, epilepsy, genetic syndromes, and previous treatment with psychoactive drugs. A diagnosis of other psychiatric disorders (e.g., autism spectrum disorder, anxiety, specific learning disorders (SLDs)) was not an exclusion criterion: 50% of the participants also had a diagnosis of an SLD, 17% of the participants also had an oppositional defiant disorder, 13% of the participants also suffered from an anxiety disorder, 8% of the participants also had autism spectrum disorder, and 13% of the participants also had a mood disorder. A total of 21% of the patient’s sample received ADHD as a single diagnosis. All participants were Caucasian, spoke fluent Italian, and had normal or corrected-to-normal vision. Familial socioeconomic status (SES) was coded according to the Hollingshead scale for parental employment [39].

#### 2.1.2. Materials

(1) Full-Scale Intelligence Quotient (FSIQ)

The FSIQ was calculated using a short form of the Wechsler Intelligence Scale for Children-III or -IV (WISC-III or -IV) [40,41,42] for all participants. Only participants with FSIQ scores higher than 80 were included in the study.

(2) Clinical and behavioral measures

Conners’ Parent Rating Scales-Revised (CPRS-R): Clinical and behavioral profiles were assessed through the Italian version of the CPRS-R [43,44], which was filled out by parents. The CPRS-R is one of the most widely used instruments for assessing symptomatology in children with ADHD, offering a measure for inattention, hyperactivity, and other behavioral domains. We considered the seven factorial-derived subscales (cognitive problems, oppositional, hyperactivity–impulsivity, anxious–shy, perfectionism, social problems, and psychosomatic) and the ADHD index scale as variables for the analysis.

Clinical Global Impression-Severity (CGI-S): Clinical severity and global functioning were also assessed by the clinician through the CGI-S [37], a one-item measure evaluating the severity of psychopathology from 1 to 7 (1 = not at all ill; 7 = among the most extremely ill patients).

Children-Global Assessment Scale (C-GAS): The clinician also compiled the C-GAS [45], which is a numeric rating scale from 1 (poor functioning) to 100 (superior functioning), to evaluate the overall functioning of a child during a specific period. The C-GAS values correspond to the following scores: 1 = 10–1; 2 = 20–11; 3 = 30–21; 4 = 40–31; 5 = 50–41; 6 = 60–51; 7 = 70–61; 8 = 80–71; 9 = 90–81; 10 = 100–91.

(3) Neuropsychological measures

Amsterdam Neuropsychological Tasks (ANT): ANT [46] is a computer-based tool for measuring the three attention networks in children and adults: alerting, orienting, and executive control. All children completed four computerized tasks, which were administered in the following order: baseline speed (BS), focused attention four letters (FA4L), shifting attentional set–visual (SSV), and sustained attention dots (SAD). BS consisted of a simple reaction time (RT) task. During the FA4L, participants had to selectively respond to one target letter among four, when it was presented in the relevant diagonal position, and to ignore it when it was displayed on the irrelevant axis. SSV was used to investigate three different cognitive dimensions: vigilance, inhibition, and cognitive flexibility. Lastly, SAD was used to assess the fluctuation of attention over time. For further details about the ANT measure, refer to our previous works [47,48].

NEPSY-Second Edition: NEPSY-II [49] is a multidomain neuropsychological battery that is designed for assessing neurocognitive abilities in children from 3 to 16 years of age. The visual attention subtest was included in this study. It evaluates the speed and accuracy with which the child is able to selectively focus and maintain attention on visual targets that are inserted within a series of distractors. The child scans a series of stimuli and points to the targets as quickly and accurately as possible.

(4) Stimulation protocol

The Emotional Continuous Performance Task (e-CPT) [36,50] was performed by children during the fNIRS recording. The e-CPT is a task that is designed to examine attentional, executive, and emotional processes during the task. In the present work, the behavioral performances on the e-CPT task were not considered for the analyses.

(5) fNIRS data acquisition

**Figure 1:**
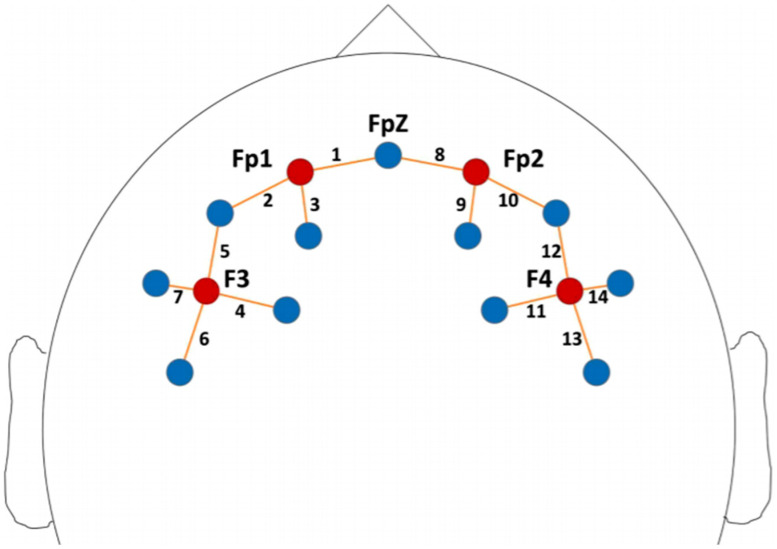
fNIRS optode localization. The probe center was positioned on Fpz and the lowest probe line was positioned along the Fp1–Fp2 line. Red circles: sources; blue circles: detectors; regions of interest: left prefrontal—source 1 (Fp1) and channels 1–3, right prefrontal—source 3 (Fp2) and channels 8–10, left frontal—source 2 (F3) and channels 4–7, and right frontal—source 4 (F4) and channels 11–14 (for further details about the fNIRS methodology used in this work, the reader is referred to Mauri and colleagues [36,53].

Details regarding the fNIRS data acquisition are described in Mauri and colleagues’ work [36]. Optode positions, source–detector combinations, and corresponding channel numbers are illustrated in Figure 1. fNIRS oxyhemoglobin (HbO) and deoxyhemoglobin (HbR) data were pre-processed using the Homer2 v2.8 software according to a validated pipeline [51,52]. Time-point concentration data were averaged across the different task conditions, thus obtaining an overall “Task” variable that measured the mean hemodynamic activation during the whole task period. fNIRS data from bilateral temporal channels were excluded from further analysis because of strong signal noise in more than 50% of participants. HbO and HbR signals were averaged in four regions of interest (ROIs) that were identified as follows: (i) left prefrontal (channels 1–3), (ii) right prefrontal (channels 8–10), (iii) left frontal (channels 4–7), and (iv) right frontal (channels 11–14). We focused the statistical analyses on the HbO data because no significant effect was previously found for HbR data [36].

### 2.2. Statistical Analysis

Statistical analyses were performed using R version 4.1.0 statistical software [54] with the additional “mclust” version 5 package for Gaussian mixture modeling [55]. The alpha level was set to 0.05 for all analyses.

Primarily, a between-group analysis was performed to characterize the group of children with ADHD compared to the TD group. More information about the between-group analysis can be found in the Supplementary Materials section (Table S1).

According to the variable distribution, a Wilcoxon signed-rank test was used as a repeated-measures analysis for evaluating the changes in clinical (C-GAS, CGI-S CPRS-R), neuropsychological (ANT and NEPSY), and NIRS variables within the group of children with ADHD after one month of MPH treatment.

Afterward, an FGMM was applied to the clinical and neuropsychological variables in which the patients showed the highest significant improvement after the MPH treatment; only subjects that were evaluated at both waves were included in the analyses. FGMMs were built through an unsupervised learning algorithm, which selected the optimal number of components from multivariate distributions [56]. The measurements obtained before and after the MPH treatment were used to conduct a bivariate FGMM to evaluate whether the within-group improvement should be considered homogeneous or heterogeneous (i.e., with more than one homogeneous subgroup within the sample with ADHD). Models estimating solutions of two or more clusters were compared using the Bayesian information criterion (BIC), with the best model having a BIC value closer to 0 [55]. The best model was selected based on the BIC and a selection heuristic based on a theoretical discussion regarding the optimal obtained solution [57]. Each subject was then assigned to their highest probability cluster. Each cluster was characterized by estimating its parameters (subgroup mean and standard deviation) for the pre- and post-treatment clinical variables. Lastly, a chi-square analysis was conducted to examine potential between-cluster differences in the psychiatric comorbidities distribution.

## 3. Results

Two participants with ADHD and two participants in the TD group were later excluded based on recruitment criteria, and four children with ADHD were excluded due to noncompliance with behavioral tasks, as mentioned in our previous work [36]. Twenty-seven children with ADHD were then evaluated. However, three patients were excluded because of technical problems during the fNIRS acquisitions. Therefore, the final sample consisted of 24 children with ADHD and 25 TD peers.

### 3.1. Between-Group Analyses

Between-group analyses were performed to characterize our sample and to identify the differences and similarities from the TD group. The between-group analyses showed no significant differences in FSIQ and SES between the ADHD group and TD, but significant differences in several clinical, neuropsychological, and neurophysiological variables. For a summary of the clinical, neuropsychological, and neurophysiological characteristics of the two samples, see Table S1 in the Supplementary Materials section.

### 3.2. Within-Group Analyses

Repeated-measures analyses were performed to detect the differences between before and after the MPH treatment. Several improvements (*p* < 0.05) were found in clinical and neuropsychological domains (Table 2). Regarding the brain hemodynamic activation, no significant HbO changes were found after the MPH administration (Table 2).

### 3.3. Clustering Analysis

#### 3.3.1. Clinical Measures

CPRS-R subscales in which the patients showed a significant improvement were considered jointly (T0 and T1) in bivariate FGMMs, separately for each subscale. Table A1 (in Appendix A) shows the BIC values that are associated with the top three ordered clustering models for each CPRS-R subscale. The BIC values of the CPRS-R cognitive problems–inattention, perfectionism, and ADHD index subscale models suggested the presence of two clusters. The clusters’ estimated results are summarized in Table 3. For the other CPRS-R subscales, the BIC suggested the existence of three clusters of subjects. These results were not deemed valid and parsimonious on a clinical and theoretical basis; hence, no FGMM was built, and the patients’ changes in oppositional, hyperactivity–impulsivity, psychosomatic problems, and social problems were considered homogeneous. Lastly, psychiatric comorbidities were not balanced across clusters in the cognitive problems subscale, with cluster 2 having a greater number of children with ADHD and comorbid SLDs. No additional differences in the distribution of psychiatric comorbidities were found.

#### 3.3.2. Neuropsychological Measures

The NEPSY and ANT scales in which the patients showed a significant improvement were considered jointly (T0 and T1) in bivariate FGMMs, separately for each subscale. Table A2 (in Appendix A) depicts the BIC values that were associated with the top three ordered clustering models for each scale.

The BIC values of the ANT–FA4L reaction time scale model suggested the presence of two clusters. Those clusters did not differ in terms of the distribution of psychiatric comorbidities within each cluster. The clusters’ estimated results are summarized in Table 4. For NEPSY–visual attention, the ANT–FA4L standard deviations of reaction time, and the ANT–SAD reaction time scales, the BIC suggested the existence of six, five, and seven clusters of subjects, respectively. These results were not considered valid and parsimonious on a clinical and theoretical basis; hence, no FGMM was built, and the patients’ changes in those dimensions were considered homogeneous. Lastly, the BIC that was associated with the models that were evaluated for the ANT–SAD standard deviations of reaction time suggested the presence of a single cluster, hence suggesting that the performance changes were to be considered homogeneous.

## 4. Discussion

The evaluation of the response to MPH treatment in ADHD is currently performed through a top-down approach, with an a priori selection of an outcome measure and an associated analysis that addresses the predictive ability of one or more other measures [15]. The main aim of the present repeated-measures observational study was to explore the utility of a bottom-up, unsupervised approach by considering clinical, neuropsychological, and hemodynamic measures to highlight the presence of distinct patterns of response to MPH in a sample of 24 school-aged children with ADHD. To achieve this goal, we tested the existence of homogeneous clusters of subjects possibly showing different responses to MPH by using an ML algorithm (FGMM). Symptoms severity, attentive performances, and brain activation were assessed before (T0) and after one month (T1) of MPH administration.

At T0, we also collected data from an age- and gender-matched sample of 25 typically developing (TD) children in order to characterize our clinical sample compared to a control group. As expected, children with ADHD presented higher psychopathological traits and worse neuropsychological performances. Moreover, differences at the neurophysiological level were found (for further discussion about the between-group differences, see the previous work published by Mauri and colleagues [36]).

Regarding the response to MPH, children with ADHD exhibited a significant improvement in clinical symptomatology after one month of MPH administration. These results were expected and in line with the literature, highlighting the well-documented effects of MPH administration [58,59,60]. Interestingly, the most severe symptoms were also those that improved the most after pharmacological treatment. As expected [61], significant amelioration was also observed in the neuropsychological profile across all cognitive tasks. In particular, in line with previous works [62,63], the reaction time variability showed a greater improvement after the MPH treatment.

Lastly, no significant change was found regarding the brain hemodynamic activation after the MPH treatment. This result was not in line with the recent review of Grazioli and colleagues [20] in which the majority of the reviewed studies indicated increased oxygenated hemoglobin concentrations after pharmacological treatment. However, it is possible that subcortical changes evoked by MPH treatment [64] could be more evident in brain regions that could not be detected by NIRS technology.

Next, in order to find possible patterns of behavioral and attentive responses to MPH, FGMM clustering analyses were implemented using clinical and neuropsychological measures that showed a significant improvement. To our knowledge, this is the first study to use an unsupervised algorithm to identify clusters of symptom improvements in children with ADHD.

Our analysis suggested the existence of two clusters of subjects in several psychopathology areas, as described by the parents using the CPRS-R. This finding confirmed the high variability in the clinical response of children with ADHD [15,17]. On the other hand, the present results suggested that the CPRS-R could be a good instrument to differentiate ADHD responses to MPH.

With specific respect to the CPRS ADHD index subscale, the results highlighted that the majority of children showed a significant improvement after the MPH treatment, with a score under the clinical cut-off, whereas a small portion of children had lesser improvements. This result could represent corroboration of previous research results addressing specificities of MPH efficacy [11,12] through a bottom-up rather than a top-down approach.

Concerning the CPRS cognitive problems subscale, the largest cluster of patients showed mean before MPH values within the clinical band and, after MPH, within the nonclinical range; the smallest cluster was represented by subjects showing both before- and after-MPH mean values within the clinical range. In this case, we observed a large improvement in both groups of subjects despite the smallest cluster showing more severe before-treatment mean scores. This result is in line with what was reported in the literature addressing improvements in the attentional domain after MPH treatment [11,12].

Regarding the perfectionism subscales, we found the biggest group showing both before- and after-MPH mean values below the clinical range and a small improvement; the smallest group showed mean values within the clinical band before the MPH treatment and a large improvement after the MPH treatment.

Lastly, FGMM clustering analyses were implemented for neuropsychological variables. Two clusters of subjects were detected regarding the ANT–FA4L RT. Specifically, the largest group showed a high reaction time before the MPH treatment and a remarkable improvement after the MPH treatment, as suggested by previous studies on visual attention capacity [65]; on the other hand, the smallest group showed a faster reaction time before the MPH therapy but a smaller improvement after the MPH treatment.

The between-clusters differences identified by our analysis did not appear to be due to the psychiatric comorbidities of children with ADHD, with the exception of differences in CPRS cognitive problems subscale. In that case, children with ADHD and comorbid SLD were more frequent (80% vs. 20%) in the cluster of children with scores in the clinical range before and after the MPH treatment. While this is not surprising because the CPRS cognitive problems subscale specifically identifies children having difficulties at school, this preliminary result suggested that SLD could negatively interfere with the treatment for the inattention domain, as previously described in recent literature [66].

The novelty of our study is represented by the use of an unsupervised ML algorithm to identify hidden clusters of patients that are characterized by specific clinical and neuropsychological modifications following pharmacotherapy. Notably, children with ADHD do not seem to constitute a single homogeneous group; on the contrary, it is likely that a single clinical diagnosis could “mask” the existence of various phenotypes of response to MPH, as evidenced by our results in terms of clinical improvement and neuropsychological performances. This is in line with the current literature that reports MPH’s efficacy to be around 70%, highlighting that approximately 30% of children do not positively respond to MPH treatment [11,67]. Hence, it is possible to hypothesize that pharmacological treatment with MPH acts on various symptom areas, and psychopathological comorbidity and neurophysiological characteristics may play a role in influencing the response to MPH. Future studies in this field considering bigger samples presenting neuropsychiatric comorbidities could disentangle these phenomena. Our research additionally suggested that the specificities that were detected regarding the symptomatology improvement following MPH treatment also characterized non-core ADHD manifestations, such as the CPRS perfectionism subscale. This result suggests deepening the exploration of other positive effects of MPH on clinical symptomatology or psychological functioning.

The main research and clinical implication that was derived from the present work is the use of a bottom-up approach in the study of responses to treatment. Indeed, the adoption of an unsupervised ML analysis rather than an a priori choice of outcome measures could offer new insights into detecting specificities in response to MPH. Indeed, clustering analysis does not require any theoretical assumption, thus offering clinical and research evidence that is useful for a personalized medicine approach.

Despite the mentioned preliminary results, some limitations must be addressed. First, our ML analysis could be highly specific to our clinical group, in consideration of the small sample size. Future investigations could employ a bigger sample size and verify the validity and direction of the present preliminary findings. Furthermore, our small sample did not allow us to fully investigate the role of psychopathological comorbidities in the different responses to medical treatment. In addition, the present study was not designed to include a second evaluation for the control group, which would have provided a possible comparison with children with ADHD after the MPH administration. Future clustering analyses could explore the differences between children with ADHD and children with other psychiatric conditions and TD groups. Lastly, our repeated-measures work had a brief duration (one month of MPH administration), which did not allow us to evaluate the maintenance of these effects over a long period.

## 5. Conclusions

In conclusion, our findings confirmed that MPH treatment was followed by clinical and neuropsychological improvements in children with ADHD, as highlighted by previous research. The novelty of our study consisted in the implementation of a rarely used analysis method, which demonstrated that clinical and neuropsychological ameliorations could be inhomogeneous among all patients and clearly indicated the existence of clusters of subjects that improved in different ways. The present ML method, if replicated in future studies with larger samples, could provide the first step toward identifying specific clusters of MPH responses. An ML approach, together with traditional statistical tools, may be promising in detecting characteristics of treatment response, and it could represent the key to exploring personalized medical necessities.

## Figures and Tables

**Table 1:** Study Design. The evaluations were performed before (T0) and after (T1, for the ADHD group) MPH intake.

	T0		T1
	ADHD Group	TD Group	ADHD Group
FSIQ	✓	✓	X
SES	✓	✓	X
Clinical	✓	✓	✓
CPRS-R	✓	✓	✓
Neuropsychological	✓	✓	✓
fNIRS	✓	✓	✓

Notes: ✓—performed; X—not performed; ADHD—attention-deficit/hyperactivity disorder patients group; Clinical—clinical severity and global functioning assessed by the clinician; CPRS-R—Conners’ Parent Rating Scales-Revised; fNIRS—functional near-infrared spectroscopy recording; FSIQ—Full-Scale Intelligence Quotient; MPH—Methylphenidate; SES—socio-economic status; T0—first evaluation; T1—second evaluation; TD—typically developing peers group.

**Table 2:** Repeated-measures analyses results. Clinical, neuropsychological, and neurophysiological characteristics of the sample before and after the treatment.

	T0	T1	Statistic Value	*p*
C-GAS Value (percentage of subjects)	4 (18%) 5 (76%) 6 (6%)	5 (13%) 6 (13%) 7 (25%) 8 (30%) 9 (19%)	0 ^a^	<0.001
CGI-S Value (percentage of subjects)	4 (18%) 5 (59%) 6 (23%)	2 (6%) 3 (19%) 4 (56%) 5 (19%)	136 ^a^	<0.001
CPRS-R (mean ± SD)				
Oppositional	72.88 ± 16.55	61.52 ± 17.11	158 ^a^	0.002
Cognitive problems	81.08 ± 11.62	70.57 ± 15.22	201.5 ^a^	<0.001
Hyperactivity–Impulsivity	77.33 ± 11.50	68.48 ± 17.06	161 ^a^	0.008
Anxious–Shy	52.25 ± 11.10	48.05 ± 10.05	98.5 ^a^	0.31
Perfectionism	53.66 ± 11.71	45.47 ± 8.28	111 ^a^	0.004
Social Problems	71.29 ± 19.89	62.90 ± 18.96	99 ^a^	0.028
Psychosomatic Problems	53.04 ± 13.80	47.00 ± 6.20	76.5 ^a^	0.032
ADHD index	81.54 ± 9.61	71.52 ± 13.70	201 ^a^	<0.001
NEPSY (mean ± SD)				
Visual Attention	9.30 ± 3.52	11.95 ± 3.57	5.0 ^a^	<0.001
ANT (ms) (mean ± SD): Baseline Speed				
RT	406.35 ± 134.82	384 ± 130.34	73.0 ^a^	0.890
SD of RT	234.30 ± 195.08	192.52 ± 160.83	75.0 ^a^	0.963
ANT (ms) (mean ± SD): Focused Attention Four Letters				
RT correct responses	1304.89 ± 491.08	1001.77 ± 318.87	147.0 ^a^	<0.001
SD of correct responses RT	558 ± 304.17	341.66 ± 173.54	146.5 ^a^	<0.001
ANT (ms) (mean ± SD): Shifting Attentional Set—Visual				
RT inhibition	270.81 ± 292.63	258. 53 ± 237.83	62.0 ^a^	0.583
RT flexibility	453.06 ± 492.93	457.43 ± 249.18	63.5 ^a^	0.221
ANT (ms) (mean ± SD): Sustained Attention Dots				
Time × Series	17.27 ± 5.96	14.65 ± 5.91	119.0 ^a^	0.009
SD	3.88 ± 1.45	3.01 ± 1.35	108.5 ^a^	0.006
Neurophysiological characteristics				
fNIRS signal (mean ± SD)				
Right prefrontal	1.43 ± 3.64	0.31 ± 2.72	64.0 ^a^	0.850
Right frontal	0.78 ± 3.70	0.31 ± 2.72	60.0 ^a^	0.670
Left prefrontal	0.95 ± 3.58	−0.88 ± 3.90	91.0 ^a^	0.252
Left frontal	1.08 ± 2.44	−0.49 ± 3.41	62.0 ^a^	0.273

Notes: ANT—Amsterdam Neuropsychological Task; C-GAS—Children’s Global Assessment Scale; CGI-S—Clinical Global Impression–Severity; CPRS-R—Conners Parent Rating Scale-Revised; fNIRS—functional near-infrared spectroscopy recording; MPH—methylphenidate; ms—milliseconds; NEPSY—Developmental Neuropsychological Assessment; RT—reaction time; SD—standard deviation; T0—first evaluation; T1—second evaluation; ^a^—Wilcoxon signed-rank test.

**Table 3:** CPRS-R clusters characteristics.

Cognitive problems	Cluster	1	2
	subjects	58%	42%
	Mean before MPH (± sd)	72.99 (±6.79)	91.93 (±6.79)
	Mean after MPH (± sd)	59.54 (±6.79)	85.95 (±6.79)
	Comorbidities	SLD: 25% *	SLD: 80% *
Perfectionism	Cluster	1	2
	subjects	57%	43%
	Mean before MPH (± sd)	43.09 (±8)	76.15 (±3.38)
	Mean after MPH (± sd)	40.41 (±8)	45.75 (±3.38)
ADHD index	Cluster	1	2
	subjects	51%	49%
	Mean before MPH (± sd)	87.03 (±6.81)	74.34 (±6.81)
	Mean after MPH (± sd)	82.85 (±6.81)	59.76 (±6.81)

ADHD—attention deficit hyperactivity disorder; CPRS-R—Conners’ Parent Rating Scale-Revised; MPH—methylphenidate; sd—standard deviation; SLD—specific learning disorder. *—significant chi-square test.

**Table 4:** Cluster characteristics in neuropsychological variables.

ANT–FA4L RT	Cluster	1	2
	subjects	63%	37%
	Mean before MPH (± sd)	1586.08 (±252)	804.52 (±94)
	Mean after MPH (± sd)	1231.25 (±252)	640.67 (±94)

Notes: ANT—Amsterdam Neuropsychological Task; FA4L—focused attention four letters; MPH—methylphenidate; RT—reaction time; SAD—sustained attention dots; sd—standard deviation.

## Data Availability

The data presented in this study are available on request from the corresponding author.

## Supplementary Materials

The following are available online at https://www.mdpi.com/article/10.3390/children8111008/s1, Table S1: Sociodemographic, clinical, and neuroimaging characteristics of the ADHD and TD samples.

## Appendix A

**Table A1:** Top three FGMMs based on the BIC criterion (clinical variables). The models that were selected as best are underlined.

CPRS-R Subscale	Model Solutions and Number of Clusters	BIC Value
Oppositional	Unequal variances, 3	−353.021
	Unequal variances, 2	−241.235
	Equal variances, 3	−241.235
Cognitive Problems–Inattention	Equal variances, 2	−325.651
	Unequal variances, 2	−328.59
	Equal variances, 3	−330.895
Hyperactivity–Impulsivity	Equal variances, 3	−339.407
	Equal variances, 2	−340.9
	Unequal variances, 2	−340.903
Perfectionism	Unequal variances, 2	−307.189
	Equal variances, 2	−309.316
	Equal variances, 1	−310.097
Social Problems	Unequal variances, 3	−349.105
	Equal variances, 6	−351.544
	Equal variances, 8	−352.87
Psychosomatic Problems	Unequal variances, 3	−253.932
	Equal variances, 2	−275.908
	Equal variances, 8	−277.611
ADHD Index	Equal variances, 2	−323.539
	Equal variances, 3	−324.537
	Equal variances, 5	−324.546

Notes: BIC—Bayesian information criterion; CPRS-R—Conners’ Parent Rating Scale-Revised; FGMMs—finite Gaussian mixture models.

**Table A2:** Top three FGMMs based on the BIC criterion (neuropsychological variables). The model that was selected as best is underlined.

Neuropsychological Scale	Model Solutions and Number of Clusters	BIC Value
NEPSY–Visual Attention	Equal variances, 6	−184.236
	Equal variances, 5	−188.422
	Equal variances, 7	−188.487
ANT–FA4L RT	Unequal variances, 2	−461.331
	Equal variances, 3	−463.189
	Equal variances, 2	−465.589
ANT–FA4L RT-SD	Equal variances, 5	−376.117
	Equal variances, 6	−377.499
	Equal variances, 4	−378.505
ANT–SAD RT	Equal variances, 7	−172.838
	Unequal variances, 9	−177.858
	Unequal variances, 3	−179.52
ANT–SAD RT-SD	Unequal variances, 1	−121.226
	Equal variances, 1	−121.226
	Unequal variances, 9	−121.613

Notes: ANT—Amsterdam Neuropsychological Task; BIC—Bayesian information criterion; FA4L—focused attention four letters; MPH—methylphenidate; FGMMs—finite Gaussian mixture models; NEPSY—Developmental Neuropsychological Assessment; RT—reaction time; SAD—sustained attention dots; SD—standard deviation.
