# Supplementary material for: Patterns of Response to Methylphenidate Administration in Children with ADHD: A Personalized Medicine Approach through Clustering Analysis
Source: Children (Basel). 2021 Nov 4;8(11):1008. doi: 10.3390/children8111008 (PMC8623097; doi:10.3390/children8111008)
Supplement: Supplementary file 1 [file children-08-01008-s001.zip › children-1382933-supplementary.pdf]

Table S1. Sociodemographic, clinical, neuropsychological and neuroimaging characteristics of ADHD and TD samples.

	ADHD (N=24)	TD (N=25)	Statistic value	*p*
**Sociodemographic characteristics**				
Female:Males	2:22	4:21	0.0 [a]	1
Age (m ± SD)	10.91 ± 2.8	10.43 ± 2.9	0.6 [b]	0.565
SES (m ± SD)	47.1 ± 12.6	53.5 ± 19.7	233.5 [c]	0.4804
WISC-IV FSIQ (m ± SD)	99.53 ± 14.1	106.1 ± 17.4	238.5 [c]	0.365
**Clinical characteristics: CPRS-R**				
Oppositional (m ± SD)	**72.87 ± 16.5**	**52.13 ± 13.6**	470.5 [c]	**<0.001**
Cognitive problems/Inattention (m ± SD)	**81.08 ± 11.6**	**47.00 ± 8.7**	566.5 [c]	**<0.001**
Hyperactivity (m ± SD)	**77.33 ± 11.5**	**48.38 ± 13.1**	535 [c]	**<0.001**
Anxious/Shy (m ± SD)	52.25 ± 11.1	48.33 ± 11.0	352.5 [c]	0.185
Perfectionism (m ± SD)	**53.66 ± 11.7**	**43.29 ± 8.6**	455 [c]	**<0.001**
Social Problems (m ± SD)	**71.29 ± 19.9**	**53.08 ± 11.5**	443.5 [c]	**<0.001**
Psychosomatic Problems (m ± SD)	**53.04 ± 13.8**	**46.17 ± 5.9**	386.5 [c]	**0.039**
ADHD index (m ± SD)	**81.54± 9.6**	**47.92 ± 9.7**	566.5 [c]	**<0.001**
**Neuropsychological characteristics**				
Nepsy - Visual Attention	9.30 ± 3.5	10.04 ± 3.2	252 [c]	0.614
**ANT** (ms) (mean ± SD)				
Baseline speed				
RT	395.29 ± 136.4	362.54 ± 113.8	227.0 [c]	0.556
SD of RT	225.53 ± 205.8	158.13 ± 119.1	242.5 [c]	0.315
Focused Attention 4 Letters				
RT correct responses	1304.895 ± 491.1	1071.435 ± 371.9	276.5 [c]	0.146
SD of correct responses RT	**558 ± 304.2**	**326.5 ± 176.4**	**324 [c]**	**0.008**
Visual set-shifting				
RT inhibition	270.81 ± 292.6	272.38 ± 268.4	192.0 [c]	1

RT flexibility	453.06 ± 492.9	506.13 ± 419.2	171.0 [c]	0.571
Sustained attention dots				
Time x Series	17.2 ± 5.9	15.4 ± 5.4	199 [c]	0.408
SD	3.8 ± 1.5	2.9 ± 1.4	235 [c]	0.053
**Neurophysiology: fNIRS**				
Prefrontal right (m ± SD)	**1.43 ± 3.6**	**3.50 ± 5.4**	104.0 [c]	**0.001**
Frontal right (m ± SD)	**0.78 ± 3.7**	**4.46 ± 4.1**	100.0 [c]	**0.014**
Prefrontal left (m ± SD)	0.95 ± 3.6	1.83 ± 4.8	176.0 [c]	0.360
Frontal left (m ± SD)	1.08 ± 2.4	0.95 ± 3.1	172.0 [c]	0.831

Notes: ADHD= attention deficit hyperactivity disorder patients group; ANT= Amsterdam Neuropsychological Task; CPRS-R= Conners Parent Rating Scale- Revised; fNIRS= functional near-infrared spectroscopy; m = mean; ms= milliseconds; NEPSY= A Developmental NEuroPSYchological Assessment; TD = Typically developing peers group; SD = standard deviation; RT= Reaction time; SES= Socioeconomic status; WISC-IV= Wechsler Intelligence Scale for Children-IV Full Scale Intelligence Quotient. a = Pearson's Chi-squared test; b = Independent two sample T test; c = Wilcoxon rank sum test.
